# Supplementary material for: Kaptive Web: User-Friendly Capsule and Lipopolysaccharide Serotype Prediction for Klebsiella Genomes
Source: J Clin Microbiol. 2018 May 25;56(6):e00197-18. doi: 10.1128/JCM.00197-18 (PMC5971559; doi:10.1128/JCM.00197-18)
Supplement: Supplemental material [file supp_56_6_e00197-18__index.html]

Supplemental material 

# Kaptive Web: User-Friendly Capsule and Lipopolysaccharide Serotype Prediction for Klebsiella Genomes

## Supplemental material

- Supplemental file 1 -

  Data Set S1 (MLST and Kaptive results for global *Klebsiella* data set)

  XLSX, 129K
- Supplemental file 2 -

  Data Set S2 (Kaptive results for additional O3 *Klebsiella* data set)

  XLSX, 12K
- Supplemental file 3 -

  Data Set S3 (MLST and Kaptive results for CG258 *Klebsiella* data set)

  XLSX, 60K
